# Limb Axis Disorder During Leg Length Discrepancy Treatment with Temporary Epiphysiodesis Using Eight-Plate Implants

**DOI:** 10.3390/jcm14010258

**Published:** 2025-01-04

**Authors:** Grzegorz Starobrat, Anna Danielewicz, Tomasz Szponder, Magdalena Wójciak, Ireneusz Sowa, Monika Różańska-Boczula, Michał Latalski

**Affiliations:** 1 Department of Paediatric Orthopaedics, Medical University of Lublin, 20-059 Lublin, Poland; starobrat@o2.pl (G.S.); michallatalski@umlub.pl (M.L.); 2 Department and Clinic of Animal Surgery, Faculty of Veterinary Medicine, University of Life Sciences, 20-612 Lublin, Poland; tomasz.szponder@up.lublin.pl; 3 Department of Analytical Chemistry, Medical University of Lublin, 20-059 Lublin, Poland; magdalena.wojciak@umlub.pl (M.W.); i.sowa@umlub.pl (I.S.); 4 Department of Applied Mathematics and Computer Science, University of Life Sciences in Lublin, 20-033 Lublin, Poland; monika.boczula@up.lublin.pl

**Keywords:** leg length discrepancy, temporary epiphysiodesis, eight plate, growth plate

## Abstract

**Background**: A common problem in pediatric orthopedics is leg length discrepancy (LLD). In adulthood, this may result in overload and degenerative changes in the lumbar spine, hip, and knee joints of the longer limb, and the fixed equinus position of the foot of the shorter limb. Surgical treatment using temporary epiphysiodesis with eight-plate implants is a minimally invasive, safe, and patient-tolerated procedure in LLD. However, publications mainly describe the effects of treatment in the form of achieved equalization and there is little information about the occurrence of secondary deformations. Our study aimed to determine the effect of temporary growth plate blocking on the final axis after treatment. **Methods**: The study was based on an analysis of radiographs recorded from 2010 to 2019 and an assessment of parameters such as MAD (mechanical axis deviation), mMPTA (mechanical medial proximal tibial angle), and M/at (mechanical axis of the tibia). **Results**: Twenty-four girls and thirty-six boys treated with eight-plate implants were included in the investigation. The duration of the treatment was 18 months (group I), 30 months (group II), and 42 months (group III). Our study revealed that the most significant differences were observed in the MAD parameter. MAD changed in a statistically significant manner across all investigated groups, for both girls and boys, regardless of the treatment duration. **Conclusions**: The treatment of LLD with epiphysiodesis using eight-plate implants influences both the anatomical axis of the bones and the mechanical axis of the limb.

## 1. Introduction

A common issue in pediatric orthopedics is leg length discrepancy (LLD). It occurs in approximately two-thirds of the general population. Around 10–12% of individuals have an LLD of up to 5 mm, while about 4% experience an LLD exceeding 10 mm [1]. True LLD (anatomical) is defined as the difference in length measured between the center of the femoral head and the center of the distal tibia that forms the ankle joint [2]. The causes of anatomical LLD can be either congenital or acquired. The most common congenital causes include conditions like hemiatrophy or hemihypertrophy, which affect the skeletal structure of the lower limb. Acquired LLD, on the other hand, can result from infections, nerve palsy, tumors, or surgical interventions [3]. The second type of LLD, functional, results from abnormal movements in the hip, knee and ankle joints. It may be caused by the disturbance of limb mechanics through contracture, the static or dynamic incorrect setting of the mechanical axis, and the shortening and/or weakening of individual muscle groups [4,5]. Differentiating between true and functional LLD relies on the accurate measurement of bone lengths, typically using a tape measure, followed by imaging techniques such as X-rays or CT scans for confirmation. In functional LLD, there are no structural differences in bone lengths. Diagnosis involves evaluating posture, gait, and pelvic alignment to identify compensatory factors causing the apparent discrepancy [6].

The difference in the length of the lower limbs may increase progressively (congenital, inflammatory), linearly (traumatic damage to growth plate), by decreasing (shortening injury—e.g., oblique fracture of the bone shaft), or be mixed (linear and progressive—e.g., Perthes’ disease) [2,7]. The unequal length of the lower limbs disturbs the statics and dynamics of the musculoskeletal system, negatively affecting the figure and gait pattern. Compensatory mechanisms that worry the parents of children with LLD appear, but they also negatively affect the musculoskeletal system.

In general, unequal limb length occurs in approximately 70% of the pediatric population, and it is mainly shortening without affecting the functioning of patients, with an LLD below 5 mm. Shortening above 10 mm occurs in approximately 4% of the population. Shortening below 10 mm requires observation or simple conservative treatment methods. Shortening a limb by more than 20 mm may significantly affect the statics and dynamics of the entire body and, consequently, its functioning. In adulthood, this may result in overload and degenerative changes in the lumbar spine, hip and knee joints of the longer limb and the fixed equinus position of the foot of the shorter limb. This group of patients often requires surgical treatment adjusted to age, etiology and the amount of shortening expected after the end of growth. In the case of LLD in the range of 10–30 mm, there is still no consensus among clinicians regarding the directions of orthopedic treatment [8]. However, in the case of pediatric patients, to protect the growing body, it is advisable to equalize or reduce the LLD difference to below 20 mm to minimize the risk of scoliosis, vertebral torsion and changes in muscle length [8,9,10].

**Figure 1:**
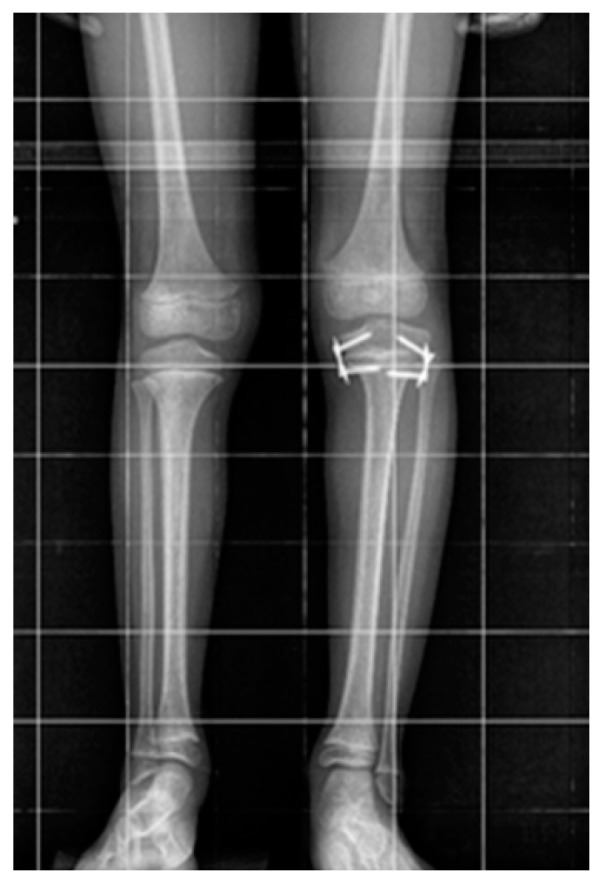
Postural X-ray of a patient treated for LLD with eight-plate implants.

Surgically blocking the epiphyseal plate (epiphysiodesis), which inhibits the growth of the longer limb, is a common method of treating limb length discrepancy (LLD). Temporary epiphysiodesis with eight-plate implants (Figure 1) is a minimally invasive, safe, and well-tolerated procedure. It provides good treatment outcomes with a relatively low rate of complications. Thus, most publications focus on achieving limb equalization, with little information available on the occurrence of secondary deformities following LLD treatment. Only a few works have highlighted the negative aspects of LLD treatment with eight-plate implants, such as a lower-than-expected degree of correction, cases of the permanent inhibition of growth plate function, and the protrusion of implants [11].

Furthermore, our study has shown that LLD treatment affects the shape of the articular surface [12]. Therefore, as a continuation of previous research, the current investigation assessed the effect of temporary growth plate blocking on the final anatomical axis of the tibia and the mechanical axis of the limb after treatment. The following parameters were examined using X-ray images obtained from 2010 to 2019 for adolescent patients treated for LLD of the lower limbs by temporary epiphysiodesis with eight-plate implants: angle between the mechanical axis of the limb and the anatomical axis of the tibia (M/aT), mechanical medial proximal tibial angle (mMPTA) and deviation of the mechanical axis (MAD).

## 2. Materials and Methods

### 2.1. Research Material

This study was retrospective in nature and was conducted based on medical records from 2010 to 2019 stored in the archives of the Pediatric Orthopedic Clinic at the Medical University of Lublin. The Bioethics Committee of the Medical University of Lublin approved the study protocol (number KE-0254/81/2020, dated 30 April 2020). The research material included radiographs of patients treated for LLD.

The cases selected for the study included patients aged 8–14 years with idiopathic leg length discrepancy, treated using temporary epiphysiodesis with eight-plate implants (inclusion criteria). Patients with LLD due to causes other than idiopathic origin, those who had undergone surgical procedures in the limb segment planned for treatment, or patients whose anatomical axis of the tibia and mechanical axis of the limb were disturbed were excluded from the study.

Finally, radiological assessments were performed for 60 patients (24 girls, 36 boys), who were divided based on the duration of their LLD treatment: 18 months of treatment (group I), 30 months of treatment (group II), and 42 months of treatment (group III). The data of the patients whose radiographs were included in the study are provided in Table 1.

### 2.2. Investigated Parameters

A full-length standing AP radiograph of the lower limbs (teleroentgenogram) was used to evaluate the angular and linear parameters. A single radiographic exposure of both lower limbs was obtained, with the radiation beam centered on the knees from a distance of approximately 180 cm. The patient stood upright with both patellas facing directly forward. All examinations were conducted using the same radiographic equipment.

**Figure 2:**
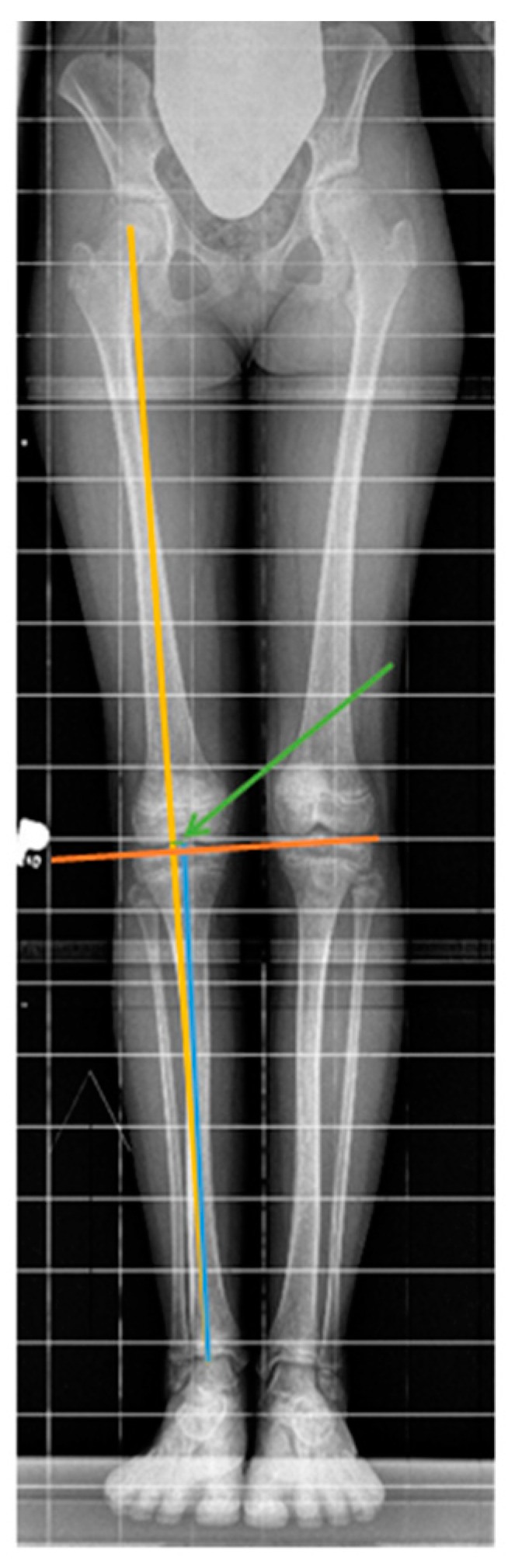
A standing X-ray image of a 10-year-old girl with leg length discrepancy (LLD) taken prior to epiphysiodesis. Graphic image of the M/aT (angle between the mechanical axis of the limb—yellow line, and the anatomical axis of the tibia—blue line), mMPTA (angle between the mechanical axis of the limb—yellow line, and the tibial joint line—orange line), and MAD (deviation of the mechanical axis from the center of the knee joint—medial or lateral—represented by a green line and indicated by a green arrow).

The parameters illustrating changes in the anatomical axis of the tibia and the mechanical axis of the limb included the following: the M/aT angle—the angle between the mechanical axis of the limb and the anatomical axis of the tibia, the mMPTA (Mechanical Medial Proximal Tibial Angle)—the angle between the mechanical axis of the limb and the joint line of the tibia, and MAD—the mechanical axis deviation (Figure 2). Measurements were conducted for the longer (treated) limb (“l”) and the shorter (non-treated) limb (“k”) based on radiographs taken every 6 months. These parameters were measured by two independent specialists, each taking three measurements, and an average was calculated from the results.

### 2.3. Statistical Analysis

For each investigated parameter, the measurements were repeated at equal intervals of 6 months. Each measurement was performed three times. Statistical analysis was conducted using Statistica 13.1 software, with the significance level set at α = 0.05. Since neither the normal distribution of the data (Shapiro–Wilk test, *p* < 0.05) nor the homogeneity of variances (Levene’s test) were confirmed, non-parametric tests were employed to assess the relationships. The Friedman test (the non-parametric equivalent to one-way ANOVA for repeated measurements) was used to compare measurements across time points, while the Wilcoxon signed-rank test was used to compare two dependent variables. Box-and-whisker plots were created to illustrate the changes.

## 3. Results

In our investigation, changes in the mechanical axis of the limb during temporary epiphysiodesis with eight-plate implants in children with LLD were examined by measuring the angle between the mechanical axis of the limb and the anatomical axis of the tibia (M/aT), mechanical medial proximal tibial angle (mMPTA) and deviation of the mechanical axis (MAD). An increase in the M/aT parameter indicates limb valgus. MAD is the line drawn from the center of the femoral head to the center of the ankle joint. In a healthy limb, it passes through the center of the knee joint, and its deviation, either medial or lateral, indicates a change in the limb axis toward valgus (positive values) or varus (negative values).

The patients included in the study were divided into three groups depending on the treatment period and the parameters were investigated every six months. At the beginning of the treatment, no disturbances were observed in the investigated parameters.

### 3.1. Changes in the Investigated Parameters After the Treatment

The statistical analysis of the differences between the values measured before and after epiphysiodesis with eight-plate implants is presented in Table 2. The greatest changes were observed in the deviation of the mechanical axis (MAD). In general, this parameter changed in a statistically significant manner in both the non-treated (k) and treated (l) legs across all investigated groups. The mechanical medial proximal tibial angle (mMPTA) changed only in boys treated for 0–18 months, with no statistically significant differences observed in the other groups. Conversely, the angle between the mechanical axis of the limb and the anatomical axis of the tibia (M/aT) changed only in girls from group III (both legs) and group II (non-treated leg), as well as in boys from group II (non-treated leg).

The statistical significance of the differences in the changes (Δ) of the investigated parameters between the treated and non-treated limbs was analyzed using the non-parametric Wilcoxon test. The results are presented in Table 3.

### 3.2. Changes in the Investigated Parameters Evaluated in Different Treatment Period

To monitor changes in the anatomical axis of the tibia and the mechanical axis of the limb over time, measurements were taken every six months. The results of the statistical analysis (*p*-values) for the treated leg versus the non-treated leg in groups I, II, and III across different treatment periods are summarized in Table 3, Table 4 and Table 5, respectively.

For group I, statistically significant differences were observed for MAD in girls during the periods of 6–12 and 12–18 months, and for mMPTA in all investigated periods, except for the first 6 months of treatment. The differences in M/at were noted only during the 6–12 month period in the girls’ group (Table 4).

In group II, changes in the parameters investigated between treated and non-treated legs were also observed (Table 5). Differences in Δ MAD were noted during the first 18 months of treatment (in the periods of 0–6 and 12–18 months in both groups). The largest differences in Δ mMPTA were found in the periods of 18–24 and 24–30 months in the girls’ group, and 0–6 and 6–12 months in the boys’ group. In turn, Δ M/AT differed in a statistically significant manner for the periods of 0–6, 18–24, and 24–30 months in the girls’ group, and 6–12 and 18–24 months in the boys’ group.

Table 6 presents data obtained for group III. Similar to group II, the most significant changes were observed for the parameter Δ MAD.

**Figure 3:**
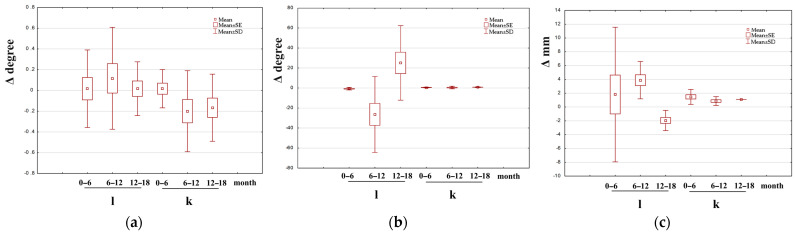
Example of box-and-whisker plots of the change in the examined parameter in group I. l—longer (treated) leg; k—shorter leg (non-treated); (**a**) M/at (the angle between the mechanical axis of the limb and the anatomical axis of the tibia) in girls; (**b**)—mMPTA (mechanical medial proximal tibial angle) in boys; (**c**) MAD (deviation of the mechanical axis) in girls.

**Figure 4:**
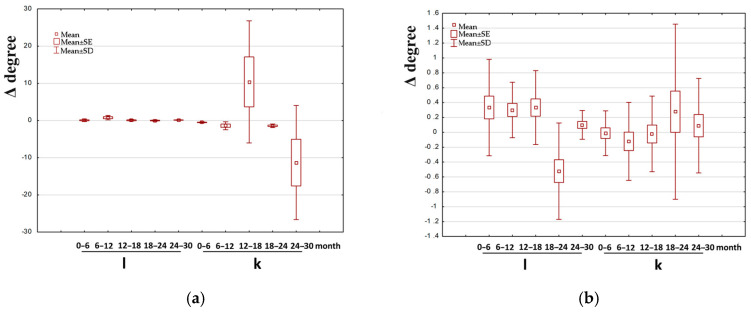

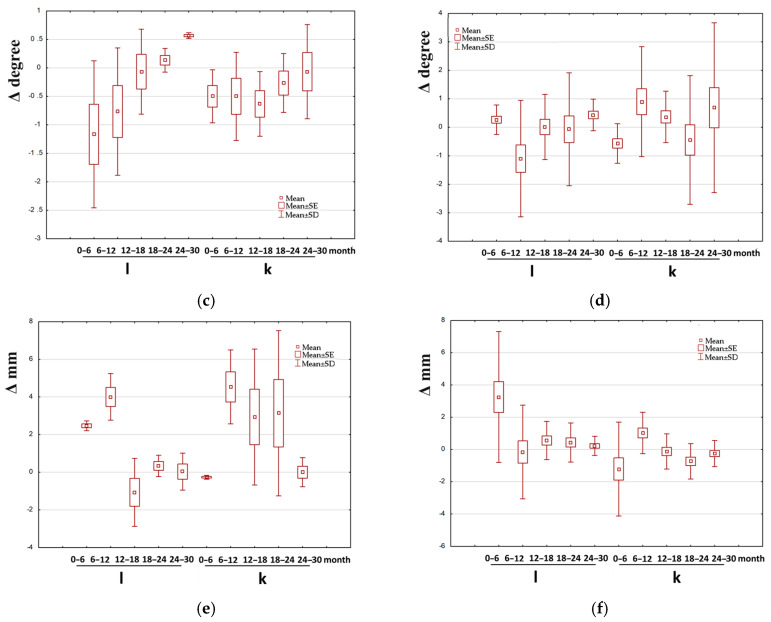
Example of box-and-whisker plots of the change in the examined parameter in group II. l—longer (treated) leg; k—shorter leg (non-treated); (**a**,**b**) M/at (the angle between the mechanical axis of the limb and the anatomical axis of the tibia) in girls and boys, respectively; (**c**,**d**)—mMPTA (mechanical medial proximal tibial angle) in girls and boys, respectively; (**e**,**f**) MAD (deviation of the mechanical axis) in girls and boys, respectively.

**Figure 5:**
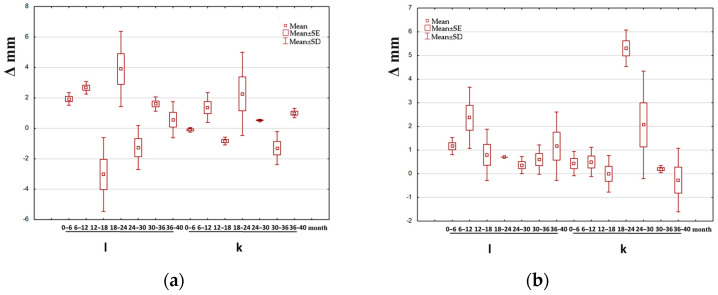
Example of box-and-whisker plots of the change in the examined parameter in group III. l—longer (treated) leg; k—shorter leg (non-treated); (**a**,**b**)—MAD (deviation of the mechanical axis) in girls and boys, respectively.

Figure 3, Figure 4 and Figure 5 show box-and-whisker plots illustrating the change in the examined parameter (Δ). Only the data for which statistically significant differences were observed between the treated and untreated leg in groups I, II, and III are presented.

## 4. Discussion

There are various methods of treating LLD depending on the severity of the discrepancy, the underlying cause, and the patient’s age. The simplest and most popular method of treating LLD is compensation with a shoe lift. This approach is easy to implement, user-friendly, well tolerated by patients, and relatively inexpensive. However, the limitation of this method lies in the extent of the discrepancy, which is typically in the range of 5–20 mm. In cases of LLD exceeding 20–30 mm, surgical methods are employed, such as the Ilizarov technique using modern external ring fixators or monolateral devices. Additionally, methods of intramedullary stabilization are used, including mechanical stabilization and advanced magnetic telescopic nails, which allow for both lengthening and stabilization. The other option is limb shortening. Acute shortening is achieved intraoperatively through bone resection, followed by forced compression. In growing children, growth can be arrested through timely surgical intervention, which involves blocking the epiphyseal plates near the knee of the longer leg [13,14].

Epiphysiodesis using eight-plate implants is a minimally invasive treatment for LLD. It is well tolerated and accepted by pediatric patients and their parents. It is often offered to patients as an alternative to long treatment with the Ilizarov method, requiring stabilizers and carrying a higher risk of complications.

The assessment of limb length discrepancy (LLD) relies heavily on radiographic analysis. Angular parameters, such as the anatomic angle and mechanical axis deviation (MAD), are crucial for evaluating limb alignment and identifying compensatory deformities. This radiographic data are indispensable for developing customized surgical treatment plans.

There are only a few scientific papers that describe both the effects of LLD treatment with temporary epiphysiodesis—equalization—and the associated complications. These complications mainly involve implant damage, such as rupture, dislodgement, or displacement. Additionally, some papers report permanent damage to the growth plate, where after implant removal, the cartilage failed to resume its function, leading to a disruption of the lower limb axis [15,16,17,18,19,20,21].

The observation of eight-plate implants by Stevens in 2007 (Orthofix GmbH, Lewisville, TX, USA) solved the main problem of temporary epiphysiodesis and permanent damage to the growth plate. The implant, which enables the gradual inhibition of growth, does not damage the growth plate. The design of the implant moves the point of support of the forces blocking the growth plate away by approximately 30 mm from its outer part, which increases the surface area of impact of these forces [22].

In our studies, no cases of permanent growth cartilage damage were observed. Patients whose lower limb equalization occurred before the end of growth had their implants removed, and follow-up examinations confirmed the continued normal growth of the operated limb.

According to Stevens’ theory, the use of eight-plate implants in symmetric epiphysiodesis exerts an uneven effect on the growth plate, depending on the distance of the cells from the implant. Chondrocyte columns located closer to the implant are more strongly inhibited, while those closer to the center of the knee joint are less inhibited. In 2013, Lauge-Pedersen et al. presented work based on the assessment of 10 patients treated for LLD using eight-plate implants. They evaluated the results of egalization at 0, 3, 6, 9, 12, 24, 52, and 80 weeks after blocking the growth plate. After 80 weeks of treatment, the equalization results ranged from 5.6 mm to 6.4 mm. The authors also reported the possibility of changing the shape of the joint as a result of the asymmetric, unpredictable effect of the eight-plate implant on the growth plate [23].

We noticed a change in the axis of the mechanical limb—MAD—in our analysis. We observed emerging varus in all groups. In group I, the increase was from 2.2 mm to 14.5 mm (mean: 12.5 mm) in 20 out of 24 patients (83.3%), in group II, the increase was from 1.5 mm to 18.3 mm (mean 14.5) in 21 of 24 patients (88.8), and in group III, the increase was from 16.4 mm to 22.7 mm (mean 19.7 mm) in 6 of 12 patients. Similar observations were reported by Gorman et al., who assessed changes in the mechanical axis based on the part of the knee joint that was blocked. They divided LLD patients treated with temporary epiphysiodesis into three groups. The first group included patients who had the distal part of the femur blocked, in the second, the proximal part of the tibia and in the third, both regions of the knee joint. Among the 54 patients, 27 (50%) experienced a shift in the mechanical axis of 10 mm toward varus in the second and third groups. The fewest changes in the KD axis occurred in patients who had only the distal part of the femur blocked. The average follow-up time was 2.8 years [18]. This is a disturbing phenomenon because the change in MAD additionally affects the biomechanics of the joint, increasing the load in the medial compartment.

One of the first to describe changes in the KD axis during temporary epiphysiodesis was Raab et al., who in 2001 described small deviations in the mechanical KD axis during the frictional treatment of LLD with Blaunt staples. It occurred in 4 out of 24 (16.6%) cases examined and was in the range of 4–9 degrees. These patients did not require secondary corrective treatment [15]. In 2014, Siedhoff presented the results of a study in which he analyzed the achieved alignment and change in the mechanical axis of the KD on anterior-posterior postural radiographs. The observation group consisted of 32 cases of patients aged from 8 to 15 years (average 12.8). However, this study included patients with larger LLD, ranging from preoperative, with 9–45 mm (mean 2.3) and expected at maturity, with 10–50 mm (mean 2.6). The treatment time was from 7 days up to 69 months (average 31). After the completion of treatment, an LLD of less than 5 mm was achieved in 10 patients, LLD below 10 mm in 21 patients, and LDD above 20 mm in only 1 patient [24]. The author assessed changes in the mechanical axis through the MAD parameter in the operated and non-operated limb. In the operated KD it was −0.06 cm (average −2.87 to 1.91), and in the non-operated KD, it was 0.4 cm (−2.31 to 2.48). The axes of both KDs differed by 0.34 cm (−2.77 to 2.78). The author considered a MAD deviation value above 1 cm to be clinically significant. In the study group, there were no KD axis disorders requiring secondary orthopedic correction.

Siedhoff’s publication is the only study that describes not only the change in the mechanical axis of the KD, but also the change in the position of the articular surfaces of the distal part of the femur, LDFA, and the proximal part of the tibia, MPTA. In his group of patients, after the completion of treatment, the LDFA angle on the operated side was on average 87.2 degrees (81–94), and on the non-operated side 86.5 degrees (82–90), a difference of 0.7 degrees (−8 to 9). The operated MPTA angle was 88.3 degrees (83–94), and in the non-operated, it was 88.1 degrees (82–92), with a difference of 0.2 degrees (−7 to 8). The described changes in the MPTA angle illustrate limb varus.

Borbas et al. [25], in a study from 2019 comparing the results of LLD treatment with final and temporary epiphysiodesis, also presented complications in the form of changes in the KD axis treated with eight-plate implants. It occurred in 2 patients out of 17 (11.7%) treated with temporary epiphysiodesis. These were axis changes in the direction of varus by approximately 5 degrees, which appeared after 12 months of LLD treatment. In the remaining patients, he did not notice any significant changes in the AC axis. We made similar observations in this work, with the difference being that a change occurred in all patients regardless of age at the initiation of treatment.

Changes in the limb axis during LLD treatment with the eight-plate implants may result from asymmetric changes in the surface of the knee joint described by Starobrat et al. In the paper, the authors present changes in the shape of the articular surface of the tibia after LLD treatment with the eight-plate method [12].

### Limitations of the Study

The research group was relatively small, though it is still larger than those in previous studies. Moreover, grouping the patients based on the duration of their treatment did not take into account the differences in the age and skeletal maturity of the patients, which could be significant for the effectiveness of the treatment. Even patients within the same chronological age group may have different skeletal ages, which can affect their growth rates and response to treatment. Additionally, the initial severity of limb length discrepancy, its underlying cause, and the presence of comorbidities can influence the time required to achieve the desired treatment outcome. The individual growth rate of each child, shaped by various genetic and environmental factors, adds further variability.

## 5. Conclusions

Our study demonstrated that the treatment of LLD with epiphysiodesis using eight-plate implants affects both the anatomical axis of the bones and the mechanical axis of the limb. However, the impact of the eight-plate implant on the sensitive growth plate is still not yet fully understood. This topic requires further research with a larger patient group, and there is a need for future long-term follow-up studies to elucidate the potential adverse effects of LLD treatment.

## Figures and Tables

**Table 1:** Detailed data of patients whose radiographs were included in the study.

Parameter	Group I (*n* = 24)		Group II (*n* = 24)		Group III (*n* = 12)	
Age y.m average/(range)	13.2/(12.2–13.8)		10.11/(10.2–11.1)		9.0/(8.6–9.6)	
LLD cm average/(range)	2.0/(1.5–2.8)		2.6/(1.4–3.6)		2.4/(1.6–3.6)	
	Boys	Girls	Boys	Girls	Boys	Girls
Number of patients	12	12	18	6	6	6
Age y.m average	13.0	13.3	11.3	10.6	9.2	8.11
Age y.m range	12.2–13.1	2.1–13.1	10.7–11.1	10.2–10.1	9.0–9.6	8.1–9.6
LLD cm average	2.1	2.0	2.6	2.6	2.5	2.4
LLD cm range	1.5–2.8	1.5–2.6	1.5–2.8	1.4–3.6	2.0–3.2	1.6–3.6

y.m—year.months; LLD—leg length discrepancy.

**Table 2:** Statistical significance of the differences (*p*) in values obtained before and after epiphysiodesis.

Parameter	G I (0–18 Months)		G II (0–30 Months)		G III (0–42 Months)	
	Girls	Boys	Girls	Boys	Girls	Boys
M/at l	0.9576	0.4784	0.5188	0.0758	0.0001	0.7236
M/at k	0.1896	0.6942	0.0026	0.0247	0.0087	0.0636
mMPTA l	0.6172	0.0042	0.7209	0.0599	0.0671	0.3032
mMPTA k	0.5582	0.0002	0.8931	0.1449	0.2271	0.5084
MAD l	0.0061	0.0012	0.0003	0.0066	0.0001	0.0001
MAD k	0.0001	0.3618	0.0001	0.1369	0.0004	0.0001

l—longer (treated) leg; k—shorter leg (non-treated); M/at—the angle between the mechanical axis of the limb and the anatomical axis of the tibia; mMPTA—mechanical medial proximal tibial angle; MAD—deviation of the mechanical axis. Red color means that value was statistically significant (*p* < 0.05).

**Table 3:** P probability values (treated leg vs. non-treated leg) for the Wilcoxon test—comparison in total time of treatment.

Group	Girls			Boys		
	Δ M/at	Δ mMPTA	Δ MAD	Δ M/at	Δ mMPTA	Δ MAD
I	0.1579	0.2094	0.8753	0.1167	0.0022	0.9375
II	0.0679	0.0678	0.0277	0.3271	0.0222	0.0582
III	0.0277	0.9165	0.0277	0.9165	0.9165	0.9165

M/at—the angle between the mechanical axis of the limb and the anatomical axis of the tibia; mMPTA—mechanical medial proximal tibial angle; MAD—deviation of the mechanical axis. Red color means that the value was statistically significant (*p* < 0.05).

**Table 4:** P probability values (treated leg vs. non-treated leg) for the Wilcoxon test—comparison in different time periods for group I.

Period	Girls			Boys		
	Δ M/at	Δ mMPTA	Δ MAD	Δ M/at	Δ mMPTA	Δ MAD
0–6 m	0.6379	0.0995	0.8753	0.0994	0.4327	0.6379
6–12 m	0.0357	0.8139	0.0228	0.8139	0.0047	0.9375
12–18 m	0.5076	0.2094	0.0022	0.1579	0.0022	0.0597

M/at—the angle between the mechanical axis of the limb and the anatomical axis of the tibia; mMPTA—mechanical medial proximal tibial angle; MAD—deviation of the mechanical axis. Red color means that value was statistically significant (*p* < 0.05).

**Table 5:** P probability values (treated leg vs. non-treated leg) for the Wilcoxon test—comparison in different time periods for group II.

Period	Girls			Boys		
	Δ M/at	Δ mMPTA	Δ MAD	Δ M/at	Δ mMPTA	Δ MAD
0–6 m	0.0277	0.2851	0.0277	0.1627	0.0209	0.0033
6–12 m	0.0679	0.6547	0.9165	0.0010	0.0209	0.1989
12–18 m	0.9165	0.1797	0.0277	0.1446	0.3139	0.0277
18-24 m	0.0277	0.0399	0.9165	0.0113	0.9527	0.0778
24-30 m	0.0277	0.0117	0.9165	0.4742	0.5147	0.1221

M/at—the angle between the mechanical axis of the limb and the anatomical axis of the tibia; mMPTA—mechanical medial proximal tibial angle; MAD—deviation of the mechanical axis. Red color means that value was statistically significant (*p* < 0.05).

**Table 6:** P probability values (treated leg vs. non-treated leg) for the Wilcoxon test—comparison in different time periods for group III.

Period	Girls			Boys		
	Δ M/at	Δ mMPTA	Δ MAD	Δ M/at	Δ mMPTA	Δ MAD
0–6 m	0.1797	0.1158	0.0277	0.9165	0.9165	0.1158
6–12 m	0.9165	0.9165	0.0277	0.9165	0.9165	0.1158
12–18 m	0.0277	0.1158	0.1158	0.4631	0.1158	0.0277
18-24 m	0.0679	0.4631	0.9165	0.1158	0.4631	0.0277
24-30 m	0.9165	0.0277	0.0277	0.4631	0.0277	0.0277
30-36 m	0.9165	0.9165	0.0277	0.1158	0.9165	0.1158
36-42 m	0.0277	0.0277	0.9165	0.1158	0.0277	0.0277

M/at—the angle between the mechanical axis of the limb and the anatomical axis of the tibia; mMPTA—mechanical medial proximal tibial angle; MAD—deviation of the mechanical axis. Red color means that value was statistically significant (*p* < 0.05).

## Data Availability

The data presented in this study are available on request from the corresponding author.
